# Supplementary figures and images for: PURPL and NEAT1 Long Non-Coding RNAs Are Modulated in Vascular Smooth Muscle Cell Replicative Senescence
Source: Biomedicines. 2023 Dec 6;11(12):3228. doi: 10.3390/biomedicines11123228 (PMC10740529; doi:10.3390/biomedicines11123228)

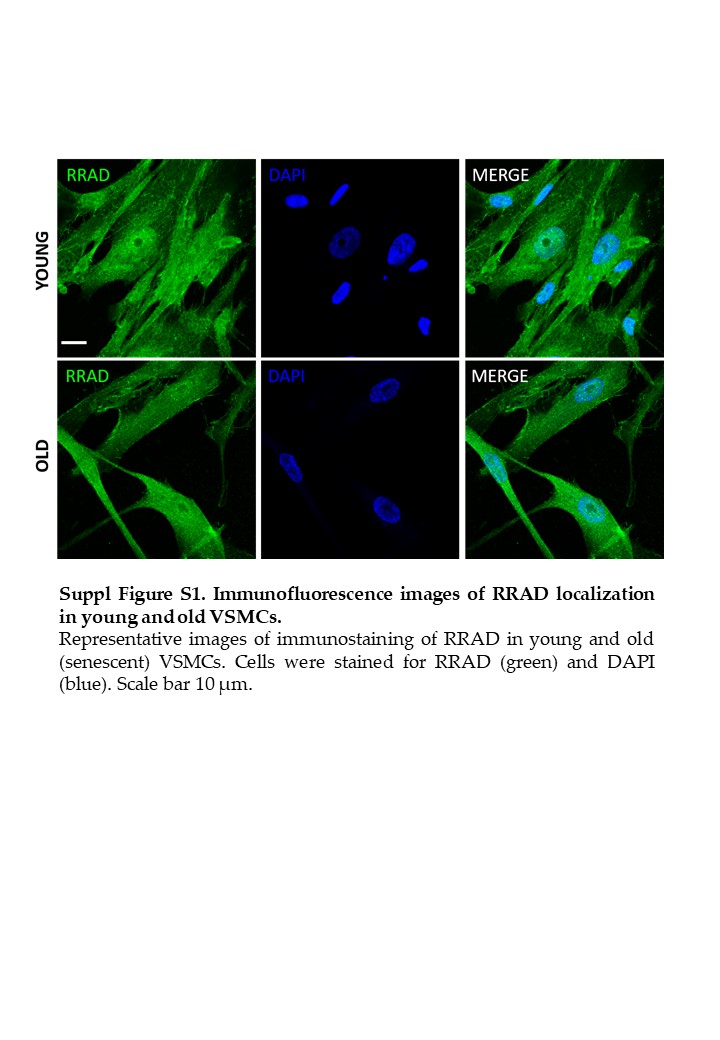

Supplement: Supplementary file 1 [file biomedicines-11-03228-s001.zip › Supplementary Figure S1.JPG]
